# Recent progress in the manipulation of biochemical and biophysical cues for engineering functional tissues

**DOI:** 10.1002/btm2.10383

**Published:** 2022-08-05

**Authors:** Behnaz Bakhshandeh, Nika Ranjbar, Ardeshir Abbasi, Elahe Amiri, Ali Abedi, Mohammad‐Reza Mehrabi, Zahra Dehghani, Cristian Pablo Pennisi

**Affiliations:** ^1^ Department of Biotechnology, College of Science University of Tehran Tehran Iran; ^2^ Department of Immunology, Faculty of Medical Sciences Tarbiat Modares University Tehran Iran; ^3^ Department of Life Science Engineering, Faculty of New Sciences and Technology University of Tehran Tehran Iran; ^4^ Department of Microbial Biotechnology, School of Biology, College of Science University of Tehran Tehran Iran; ^5^ Regenerative Medicine Group, Department of Health Science and Technology Aalborg University Aalborg Denmark

**Keywords:** biochemical cues, biophysical cues, differentiation, induction factors, tissue engineering

## Abstract

Tissue engineering (TE) is currently considered a cutting‐edge discipline that offers the potential for developing treatments for health conditions that negatively affect the quality of life. This interdisciplinary field typically involves the combination of cells, scaffolds, and appropriate induction factors for the regeneration and repair of damaged tissue. Cell fate decisions, such as survival, proliferation, or differentiation, critically depend on various biochemical and biophysical factors provided by the extracellular environment during developmental, physiological, and pathological processes. Therefore, understanding the mechanisms of action of these factors is critical to accurately mimic the complex architecture of the extracellular environment of living tissues and improve the efficiency of TE approaches. In this review, we recapitulate the effects that biochemical and biophysical induction factors have on various aspects of cell fate. While the role of biochemical factors, such as growth factors, small molecules, extracellular matrix (ECM) components, and cytokines, has been extensively studied in the context of TE applications, it is only recently that we have begun to understand the effects of biophysical signals such as surface topography, mechanical, and electrical signals. These biophysical cues could provide a more robust set of stimuli to manipulate cell signaling pathways during the formation of the engineered tissue. Furthermore, the simultaneous application of different types of signals appears to elicit synergistic responses that are likely to improve functional outcomes, which could help translate results into successful clinical therapies in the future.

**Abbreviations**
ADSCs: adipose‐derived stem cells
BDNF: brain‐derived neurotrophic factor
bFGF: basic fibroblast growth factor
BMPs: bone morphogenic proteins
CS: chitosan
ECM: extracellular matrix
EGF: epidermal growth factor
FDA: Food and Drug Administration
FGF: fibroblast growth factor
GDNF: glial cell line‐derived neurotrophic factor
GMP: good manufacturing practices
HA: hyaluronic acid
IL: interleukin
miRNA: microRNA
mRNA: messenger RNA
MSCs: mesenchymal stem cells
NGF: nerve growth factor
PCL: poly caprolactone
PDGF: platelet‐derived growth factor
PEMFs: pulsed electromagnetic fields
PLGA: poly lactic‐co‐glycolic acid
PLLA: poly l‐lactic acid
TE: tissue engineering
TGF‐β: transforming growth factor‐beta
TNF: tumor necrosis factor
USWs: ultrasound standing waves
VEGF: vascular endothelial growth factor

## 1 INTRODUCTION

Tissue engineering (TE) is a multifaceted and interdisciplinary field that is supported by the broad principles of engineering, natural sciences, and biology. The goal of TE is to develop functional substitutes for a broad wide of biomedical applications, including tissues for repairing damaged organs and in vitro models for pharmacological research. One of the key elements for the success of any TE approach is the creation of a native‐like microenvironment for the cells to recapitulate the processes occurring during development and under physiological and pathological conditions.^1^ During these processes, for instance, angiogenesis, inflammation, and wound healing, the fate of the cells is tightly controlled by several biochemical and biophysical signals from the cell microenvironment.^2^ Therefore, TE approaches have exploited inductive molecules, bioactive ligands, mechanical stimulation, and stiffness gradients to efficiently manipulate cell adhesion, migration, and lineage specification.^3^, ^4^ While significant progress has been obtained in recent years, a fascinating challenge in TE remains the accurate control of the microenvironmental cues that modulate cell fate.^5^, ^6^ Since the signaling networks that determine cell fate decisions are multiple and complex, the implementation of an appropriate natural‐like milieu requires careful consideration of the cross‐talk between the various pathways regulated by biochemical and biophysical cues. As studies have shown, the combination of biochemical and biophysical induction signals can be instrumental for efficient tissue regeneration and repair.^7^, ^8^ In this direction, there has been an increasing interest in the simultaneous application of biochemical and biophysical induction factors to synergistically promote the efficiency of TE approaches.

This review aims to summarize the main types of biochemical and biophysical stimulating factors, effective approaches, and related concepts to manipulate cell fate decisions in TE. Additionally, the review presents and discusses current advances regarding the synergistic application of these induction factors for engineering functional tissues.

## 2 BIOCHEMICAL FACTORS

**FIGURE 1:**
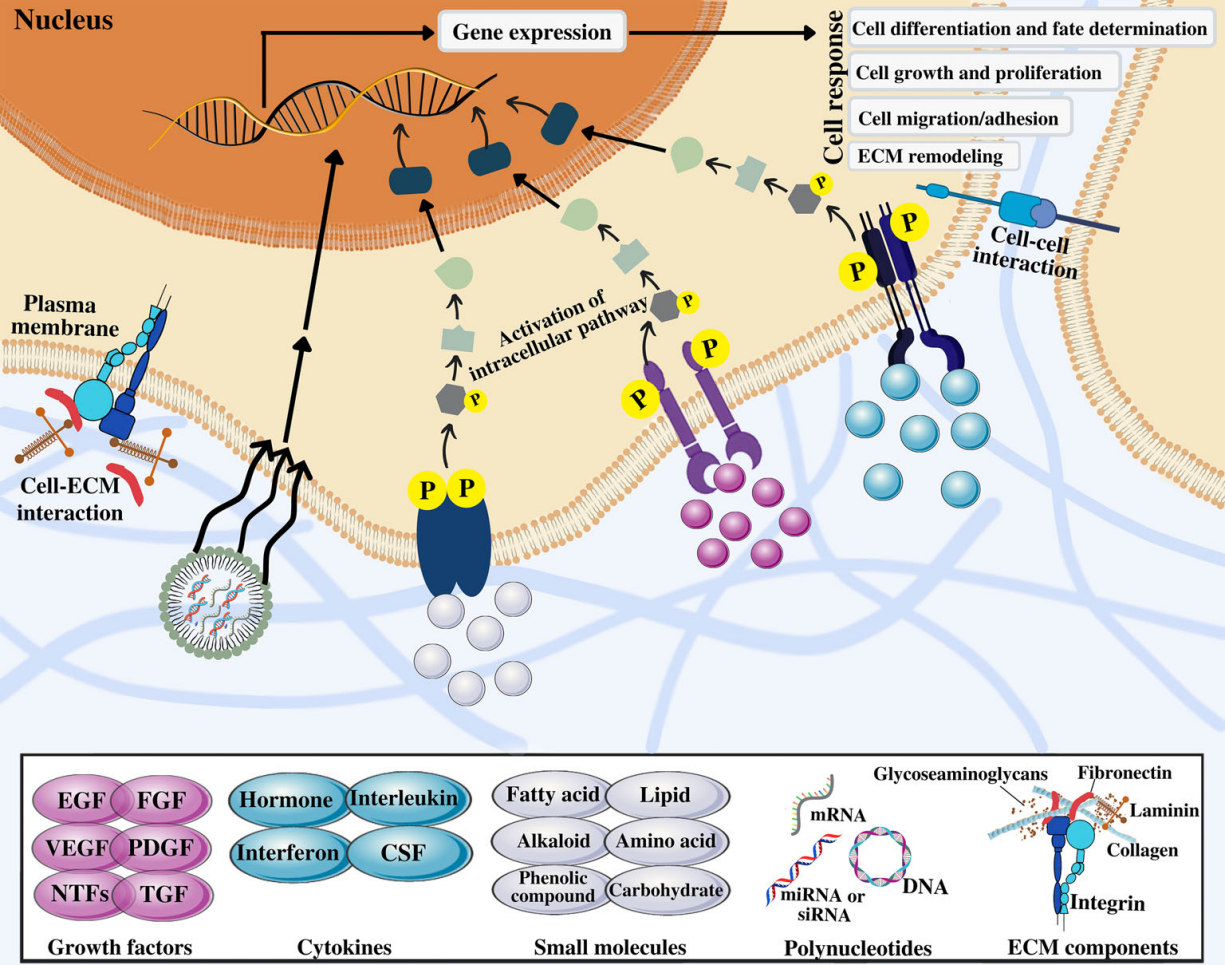
Biochemical stimulating factors involved in manipulating cell fate decisions in tissue engineering. Biochemical factors including growth factors, cytokines, small molecules, polynucleotides, and ECM components induce activation of various downstream regulatory molecules in intracellular signaling pathways which alter gene expression and subsequently lead to fate determination and different cell responses such as differentiation, growth, proliferation, migration, and ECM remodeling. CSFs, colony‐stimulating factors; EGF, epidermal growth factor; FGF, fibroblast growth factor; NTFs, neurotrophic factors; PDGF, platelet‐derived growth factor; TGF, transforming growth factor; VEGF, vascular endothelial growth factor

Biochemical induction factors include growth factors, cytokines,^9^, ^10^ small molecules,^11^ polynucleotides,^12^ and other bioactive agents. Growth factors are a superfamily of peptides that regulate a large amount of cell signaling processes and mediate cell proliferation, migration, and differentiation.^13^ Cytokines are a group of proteins creating a more stimulating microenvironment for better integration of implantable systems.^14^ A wide range of small molecules regulates many intracellular processes, resulting in changing the transcription of specific genes, and subsequently, the expression of various proteins.^15^ Unlike growth factors and cytokines, the extremely small size of small molecules leads to easy penetration through the cell membrane and affects intracellular signaling pathways.^15^, ^16^, ^17^ Due to the participation of ECM proteins in cell fate determination mediated by cell–ECM interactions, the investigation of the ECM‐derived peptides and motifs as exogenous agents provides a great opportunity for mimicking the natural cell environment.^3^ Other advanced techniques to control the level of soluble signaling molecules in TE approaches include introducing polynucleotides that alter the gene expression of selected signaling molecules on target cells.^12^, ^18^, ^19^, ^20^ The main types of biochemical factors are presented and discussed in the following subsections and summarized in Figure 1.

### 2.1 Growth factors

Growth factors are polypeptides secreted by a wide range of cell types that can stimulate or inhibit cellular functions such as proliferation, differentiation, gene expression, and migration.^21^ Scientists have identified hundreds of growth factors that have been classified into 20 families and superfamilies depending on their structural similarities.^22^ When the growth factor binds to its cell surface receptor, the signal will amplify and transfer through the cell membrane and eventually change cell function via modifying gene expression.

The action of growth factors is dependent on many factors like concentration, duration, cell location, and cell cycle state.^23^ Although using growth factors at optimal concentrations is critical for efficient tissue regeneration, their short half‐life, poor stability, enzymatic inactivation under physiologic conditions, and toxicity in high doses are the major obstacles that raise serious concerns for their clinical applications. On the other hand, direct in vivo injection of biochemical factors or supplementation of soluble growth factors to the in vitro culture medium result in some side effects; therefore, researchers simultaneously use integrated methods or regulatory agents to release them under control in damaged tissue. Even though designing the appropriate delivery system for growth factors is a challenging process, there are some state‐of‐the‐art strategies that provide the opportunity for precise and spatiotemporal control of growth factors release. Some strategies that hold great promise for the controlled release of growth factors include incorporating growth factors in the hydrogel matrix, hydrophilic materials, compositions, microencapsulation, physical or chemical surface immobilization, and triggered delivery.^24^, ^25^, ^26^

#### 2.1.1 Epidermal growth factor

Epidermal growth factor (EGF) is a 53 amino acid mitogenic polypeptide that can affect the rate of wound healing.^27^ The ERK/MAPK pathway controls gene expression, cell cycle, and proliferation, and the PI3K/Akt and JAK/STAT pathways, which trigger a range of anti‐apoptotic and pro‐survival signals and are the major intracellular signaling pathways activated by EGF signal transduction.^28^ Specifically, binding the EGF receptor to its ligand activates the receptor's tyrosine kinase activity, which stimulates downstream signaling cascades such as Ras activation and mitogen‐activated protein kinase (MAPK) activation.^29^ Subsequently, the receptor is internalized by clathrin‐coated endocytosis and EGF enters the cell to exert its effects (e.g., activation of stem cell proliferation and migration).^29^ Activation of the EGF receptor or HER‐1 leads to increased production of specific proteins related to the endothelium, keratinocytes, and corneal epithelial differentiation in vivo and in vitro.^30^, ^31^ Another role of EGF on mesenchymal stem cells (MSCs) has been reported in partial wounds and surgical incisions by increasing proliferation and subsequently improving the tensile strength of the dermis.^32^ In another study, a polylactic acid and glycolic acid (PLGA) nanofiber membrane containing EGF and Aloe vera extract promoted re‐epithelialization and wound healing.^33^ Evaluation of EGF‐loaded PLGA nanofibers in another study showed that the controlled release of encapsulated EGF from this core–shell structure can effectively accelerate the proliferation of human fibroblasts.^34^ Recent studies have shown that a combination of gelatin methacryloyl hydrogel (GelMA) with electrospun EGF‐loaded poly(3‐hydroxybutyrate‐co‐3‐hydroxyvalerate) (PHBV) promotes cell proliferation, migration, and angiogenesis. The researchers proposed this gelMA/PHBV/EGF patch as a promising tool for diabetic wound healing.^35^ Besides, several studies have found that EGF serves as a critical growth factor during satellite cell myogenesis and the formation of sarcomeric structure. Wroblewski et al. exposed scaffold‐free skeletal muscle units with EGF, enhancing skeletal muscle cell differentiation and promoting contractile function.^36^

#### 2.1.2 Fibroblast growth factor

Fibroblast growth factor (FGF) family consists of more than 20 members involved in various biological functions such as morphogenesis, brain patterning, and muscle regeneration. Also, FGFs influence various cell types like fibroblasts, astrocytes, endothelial cells, chondrocytes, and smooth muscle cells.^37^ The interaction between FGFs and their receptors activates downstream signaling pathways like RAS‐MAP and PI3K‐AKT.^38^ Two important members of this family, FGF‐1 (16 kDa) as an acidic fibroblast growth factor and FGF‐2 (17 kDa) as a basic fibroblast growth factor (bFGF) have been well fully characterized.^39^ Due to the rapid degradation of injected bFGF in the body,^40^ it should bind to heparin‐Sepharose beads for a long time release. It is reported that high doses of FGF and heparin increased the DNA replication of the fibroblasts.^41^ Also, bFGF can regulate blood vessel formation and has an important function in angioblasts induction.^42^ Among this family, FGF‐2, FGF‐9, and FGF‐18 play major roles in bone formation.^43^ Incorporation of FGF‐18 into chitin–PLGA/CaSO4 hydrogel is a promising technique for craniofacial bone defect regeneration since it has shown great improvements in bone formation.^44^ In another study, researchers designed a scaffold‐based delivery system for FGF‐2 to promote bone regeneration. They fabricated FGF‐2 loaded poly 2‐hydroxyethyl methacrylate/trimethylolpropane trimethacrylate beads and then encapsulated them into a resin. Their results showed an increase in osteoblasts proliferation and bone regeneration in calvaria defects in animal models, suggesting this platform is an effective delivery system for growth factor delivery and TE applications.^45^

#### 2.1.3 Transforming growth factor‐β

Transforming growth factor‐β (TGF‐β) is an important protein that regulates cell differentiation, proliferation, and metabolism of ECM proteins. TGF‐β superfamily consists of three main groups including the TGF‐β, the bone morphogenic proteins (BMPs), and the activins.^46^ The active form of TGF‐β (25 kDa) is a homodimer with disulfide bonds.^47^ TGF‐β signaling pathway is regulated by its bioavailability and releasing process into ECM through a multi‐steps proteolytic procedure. Also, it has an important role in regulating inflammatory responses.^48^ Until now, many functions of TGF‐β, such as stimulation of mesenchymal cells, inhibition of ectodermal cells, controlling fibrosis‐related chronic inflammatory diseases, improvement of tissue healing, and autoimmune diseases suppression, have been indicated.^49^ The impressive enhancement in matrix synthesis via endothelial cells and vascular smooth muscle which were seeded on a hydrogel, has been detected under the TGF‐β treatment.^50^

Other members of the TGF‐β superfamily are BMPs. Until now, more than 40 BMPs have been identified. These hydrophobic acid glycoproteins play different biological roles during morphogenesis and embryonic patterning. Also, they can orchestrate tissue formation throughout the body. MAPK, Smad, STAT, ERK 1/2, and PI3K‐PKB pathways are some of the cell signaling pathways regulated by these factors in the regeneration and repair of damaged tissues. Besides, in bone and cartilage TE, BMPs are extensively employed.^10^, ^51^ BMP‐2, BMP‐4, and BMP‐7 are the most widely used as osteogenic induction agents for stimulation of bone regeneration in fractures and ectopic site repair.^52^ BMP‐2 is an osteoinductive factor that enhances the proliferation, migration, and differentiation of MSCs and the endochondral ossification process.^53^ Besides, it can potentially stimulate angiogenesis. However, the natural short half‐life of this factor is an important limitation for its clinical application. To circumvent this issue, some studies suggest applying the scaffold‐based system for its delivery. In one study, an unmodified and modified nanofibrous polycaprolactone (PCL) scaffold with sulfated chitosan (CS) for the prolonged releasing time of BMP‐2 was investigated. The authors demonstrated a considerable increment in releasing time of BMP‐2.^54^ In another study, Chiu et al. compared the function of surface‐modified PCL scaffold with heparan sulfate/perlecan as a platform for sustained release of BMP‐2.^55^

Duruel et al. developed a CS/alginate/PLGA hydride scaffold loaded with insulin‐like growth factor (IGF) and BMP‐6 to examine the effect of the sequential release of these factors in periodontal regeneration. The results demonstrated a great improvement in proliferation and osteogenic differentiation of cementoblasts and suggested a new platform for efficient periodontal regeneration.^56^

#### 2.1.4 Platelet‐derived growth factor

Platelet‐derived growth factor (PDGF) is a growth‐promoting factor for fibroblasts that consists of two disulfide‐linked peptide chains that can be formed as homodimers (PDGF‐AA and PDGF‐BB) or as heterodimers (PDGF‐AB).^57^ The interaction of PDGF with various ECM and plasma proteins regulates its biological functions.^58^ The biological half‐life of intravenously injected PDGF is less than 2 min.^22^ PDGF leads to initiating a biological response via binding to its receptor, activation of protein tyrosine kinase, and subsequent generation of phosphorylation‐mediated signals.^59^, ^60^ PDGF can promote tissue regeneration by increasing the production of autocrine factors, fibronectin, and hyaluronic acid.^61^ It also plays an important role in bone regeneration and can effectively increase cartilage formation.^62^ Lee et al. immobilized PDGF‐coated poly l‐lactic acid (PLLA) fibers in spheroids of adipose‐derived stem cells (ADSCs) to investigate the effect of this controlled‐release strategy on promoting osteogenic and endothelial differentiation of ADSCs. Subsequently, in vivo experiments demonstrated enhanced bone regeneration and new vessel formation in the mouse calvarial defect model.^63^

#### 2.1.5 Vascular endothelial growth factor

Vascular endothelial growth factor (VEGF; 38.2 kDa) has an influence on the migration, proliferation, and growth of vascular endothelial cells.^23^ VEGF as a master regulator of blood vessel formation can effectively initiate morphogenesis, promote the first vascular pattern, and increase endothelial precursor cell population in vivo.^64^ Creating blood vessel networks is an essential and challenging step in TE as the diffusion of nutrients and oxygen is a major limit to the complexity and size of engineered constructs.^65^ As an important factor for forming blood vessels and vascular permeability, VEGF has recently been introduced as a therapeutic factor for tissue repair.^66^, ^67^ Tsao et al. proposed a dual growth factor delivery system including porous PLGA/silica nanoparticles loaded with VEGF and PDGF and electrospun gelatin patch. Sequential delivery of these factors promoted localized neovascularization.^68^ Park et al. reported a multi‐head 3D bioprinting method for heterogeneous TE applications. They represented a 3D‐printed prevascularized platform combined with human dental pulp stem cells for the spatiotemporal release of BMP‐2 and VEGF. After 28 days of grafting into rat cranial bone defects, the synergetic effect of BMP‐2 and VEGF considerably enhanced the regeneration of vascularized bone.^69^ In another example, a gelatin/alginate/β‐TCP scaffold was fabricated with 3D‐printing technique with subsequent entrapment of VEGF‐loaded PLGA microspheres. Supporting cell viability and proliferation, this delivery system was suggested as a novel platform for craniofacial TE.^70^

#### 2.1.6 Neurotrophic factors

Neurotrophic factors (NTFs) are endogenous soluble proteins responsible for regulating cellular growth, survival, proliferation, re‐myelination, migration, and morphological plasticity. Nerve growth factor (NGF), glial cell line‐derived neurotrophic factor (GDNF), brain‐derived neurotrophic factor (BDNF), neurotrophin‐3 or neurotrophin‐4, and ciliary neurotrophic factor are some of the most well‐known neurotrophic factors that are extensively used in neuronal regenerative medicine. These factors modulate intracellular signaling pathways through specific neurotrophic receptors such as tyrosine receptor kinase and p75 receptor.^71^ Due to in vivo short half‐life of neurotrophic factor, sustained scaffold‐based delivery strategies could enhance the regeneration capacity of nerve defects.

NGF plays an essential role in the growth and developmental plasticity in the vertebrate peripheral and central nervous system resulting in its vast applications in neural TE.^22^, ^72^, ^73^, ^74^, ^75^ For example, encapsulation of NGF into chitosan nanoparticles (NGF‐nCS) effectively promoted the trans‐differentiation into neuron‐like cells.^76^ In another study, immobilization of NGF and BDNF on the NH_2_^+^/heparin bioactive surfaces induced neurite outgrowth in dorsal root ganglions in vitro.^77^ Xia et al. loaded VEGF and NGF into the core–shell PLLA electrospun scaffold. Sequential release of NGF and VEGF enhanced the neural differentiation of stem cells in vitro. Furthermore, postoperation experiments showed neovascularization along with nerve regeneration in the rat sciatic nerve model.^78^ Another in vitro investigation illustrated that immobilization of NGF via polydopamine‐coated PCL/CS scaffold improved adhesion, proliferation, and neurite outgrowth of PC12 cells.^79^ Lackington et al. assessed the nerve regeneration capacity of biodegradable nerve conduits incorporating NGF and BDNF in PLGA microparticles. This dose‐dependent delivery system could effectively promote neurite and axonal outgrowth and Schwann cell migration in vitro. Besides, significant enhancement in functional nerve recovery in sciatic nerve defect of animal models was confirmed its therapeutic potential for peripheral nerve repair.^80^

Hu et al. developed a thermoresponsive heparin‐poloxamer hydrogel for the controlled release of NGF and bFGF into the lesioned spinal cord. In vitro and in vivo studies showed excellent enhancement of axon regeneration and functional recovery significantly along with decreased neurons apoptosis and glial scar formation. This neuroregenerative effect was related to PI3K/Akt and MAPK/ERK signaling pathways.^81^

Zhang et al. designed collagen/β‐tricalcium phosphate conduits combined with NGF to bridge a nerve gap in vivo. Electrophysiological and histomorphometric tests demonstrated that this biomedical system could improve functional recovery and axonal regeneration.^82^ Taken together, these experiments confirmed the potential application of NGF and other neurotrophic factors, especially in peripheral TE.

### 2.2 Cytokines

Cytokines are secreted factors that modulate cell growth, differentiation, and immunity. Usually, they are classified into two types: type I includes hormones, interleukins (ILs), neurotrophic factors, and colony‐stimulating factors, whereas interleukin‐10 and interferons are categorized in type II.^83^ Cytokine‐secreting cells are usually related to the immune system and are often released by T cells and macrophages in the damaged area.^14^ The most effective cytokines in TE include ILs and tumor necrosis factor (TNF).^10^, ^14^ In the first 24 h after tissue damage, cytokines, including ILs (1–6), are first activated by macrophages and begin the regeneration process by re‐calling the related angiogenesis factors. In the early stages, the TNF‐α as a cytokine facilitates the regeneration process by collecting and destroying damaged parts and attracting more stem cells to the site. These factors help regenerate damaged tissue in three stages: inflammation, angiogenesis, and finally transformation with new cells.^10^ Mountziaris et al. designed a microfibrous PCL mesh containing TNF‐α to investigate the effect of TNF‐α on osteogenic differentiation of MSCs. The results demonstrated that temporal patterns of TNF‐α delivery supported the bone regeneration process.^84^ In another study, an IL‐4‐loaded hybrid bilayer scaffold was fabricated to examine the anti‐inflammatory effect of IL‐4 on articular cartilage and subchondral bone repair. Both in vitro and in vivo experiments revealed decreased inflammatory effects of IL‐1β and macrophages on chondrocytes resulting in better osteochondral regeneration.^85^

### 2.3 ECM components

The ECM provides a microenvironment that fully supports the structure and function of the tissue and meets its biological and physical needs. The ECM is produced and maintained by the cells in a dynamic equilibrium.^86^, ^87^ Investigations have shown that ECM proteins such as elastin, collagen, laminin, and fibronectin are fundamental for the survival and growth of many cell types. Collagen is the most abundant protein found in mammalian organisms, playing an important function in providing tensile strength to various tissues and supporting the function of multiple cell types. With its triple helix structure, collagen can significantly affect key cellular functions such as adhesion, proliferation, and migration.^88^ Fibronectin can enhance cell adhesion through its various binding domains.^89^ Some studies demonstrated that fibronectin‐conjugated scaffolds could effectively improve cell adhesion and infiltration depth.^90^

Hyaluronic acid (HA), as an ECM glycosaminoglycan, participates in wound repair and cell migration. The biocompatibility, biodegradability, hydrophilicity, and limited immunogenicity of HA make it an ideal choice for cartilage, bone, vascular, and skin TE.^91^, ^92^ Park et al. produced an injectable HA hydrogel loaded with a BMP‐2 mimetic peptide for osteoinduction of human dental pulp stem cells.^93^ Choi et al. revealed that encapsulated chondrocytes in HA/agarose hydrogels provided an enhanced microenvironment for chondrogenesis.^94^ As an articular cartilage‐specific proteoglycan, Aggrecan is related to the load‐bearing properties of cartilage. Ingavle et al. designed an interpenetrating network hydrogels containing aggrecan and chondroitin sulfate as bioactive signals to enhance encapsulated chondrocytes performance.^95^

Due to the appropriate biocompatibility and biodegradability of fibrin, it serves in cell delivery platforms. Montalbano et al. formulated a thermo‐responsive collagen/alginate/fibrin hydrogel to mimic the native ECM microenvironment for reconstitution of pseudo‐islets. Their results offered a tunable composite for pancreas TE and musculoskeletal regeneration.^96^

Although purified ECM proteins can be utilized for TE applications,^97^ the production of peptide sequences is simpler and more cost‐effective as compared to full‐length proteins. RGD, IKVAV, YIGSR, DGEA, PHRSN, and PRARI peptides, as modifier peptides, are usually derived from structural ECM protein domains particularly collagen, laminin, fibronectin, and elastin.^4^ Considering good solubility and stability as compared to full‐length ECM proteins, peptide sequences have been widely employed to mimic the natural ECM.^98^, ^99^ These biomolecules can effectively promote various cell functions and stimulate specific signaling pathways due to their specific signature derived from the ECM proteins.

Among the mentioned peptides, the RGD motif (Arg‐Gly‐Asp) derived from collagen, fibronectin, laminin, gelatin, vitronectin, fibrinogen, osteopontin, and sialoprotein, specifically targets a number of integrin receptors.^100^, ^101^ Integrins are heterodimeric transmembrane complexes consisting of two subunits, the α‐chain and the β‐chain, which are present on cell membranes and associated with various components of the ECM.^102^, ^103^ The binding of cells to the ECM through the α‐chain of integrins at focal adhesion sites is mediated by calcium and is essential not only for cell‐ECM adhesion but also for cell–cell adhesion.^102^, ^103^ Integrin–ligand binding mediates the interaction between external forces and the actin cytoskeleton, leading to the clustering of cytoplasmic proteins (such as FAK, vinculin, or talin) and regulating intracellular signaling cascades such as the MAPK pathway, which influence important processes including proliferation, differentiation, and migration.^104^ RGD‐modified materials have been used in cancer therapy, TE, and regenerative medicine studies.^100^, ^102^ For example, Wang et al. investigated the synergistic effect of topographic cues with peptide presentations (RGD/YIGSR) as a new approach to scaffold design for skeletal muscle TE. This study showed that surface‐mediated PLGA scaffolds can significantly affect myoblast proliferation and differentiation.^105^ Another important integrin‐binding peptide motif is the IKVAV (Ile‐Lys‐Val‐Ala‐Val), which is derived from laminin and can act as a biofunctional epitope.^106^ Cheng et al. reported that RADA16‐IKVAV self‐assembling peptide hydrogel could be used as a gap filler in nerve injury by positively affecting surviving neural stem cells and reducing glial astrocyte formation.^107^ In another study, the incorporation of IKVAV and RGD peptides into a protein nanostructured scaffold provides the chemical and structural support essential for the myogenic differentiation of the C2C12 cells.^108^ Besides, many studies focused on the effect of laminin‐derived IKVAV epitope on neuronal differentiation and neurite extension, which is a key point for neural TE.^109^

### 2.4 Polynucleotides

Due to the high cost of exogenous factors and their potential cytotoxic effects, the introduction of DNA or RNA molecules into the cells is applied for gene expression manipulation. These methods can also be used to achieve sustained gene expression in the stem cells, with the aim of improving their properties for therapeutic applications.^110^, ^111^ In this context, the role of genetically modified cells in restoring the normal function of various types of tissues has been explored by several research groups. There are a number of proteins that can be overexpressed to induce a specific cell phenotype, such as FGF‐2, BMPs, TGF‐β, IGFs (insulin‐like growth factors), VEGF, and PDGF.^112^, ^113^

The improved survival, proliferation, and metabolism of these engineered cell lines have opened the perspective of translating preclinical studies into efficient and safe therapies for numerous diseases and deficiencies, including multiple sclerosis, Parkinson's disease, heart disease, kidney injury, bone and cartilage disorders, and amyotrophic lateral sclerosis.^114^, ^115^ In addition, there have been several studies on cancer therapy based on transformed stem cells, which have been shown to reduce tumor growth and increase patient survival in various malignancies including melanomas, brain, liver, and breast tumors.^112^

Approaches for introducing genes into the desired cells are generally divided into nonviral methods (e.g., liposomes and polycations) and viral methods (e.g., adeno‐associated viruses, retroviruses, herpes viruses, and others). Each approach offers advantages and disadvantages that must be carefully considered. For example, viral delivery allows for sustained expression and highly efficient transfection, but may also trigger immune responses. On the other hand, nonviral gene transfer vectors are easy to produce, safer than the viral methods, and result in more transgenes. But, some nonviral polymeric vectors, such as polycations, may display toxicity and low efficiency. Nevertheless, nonviral vectors have lower immunogenicity and cytotoxicity, making them the method of choice in most studies.^7^, ^111^, ^112^

Despite the clear potential of gene transfer techniques, the clinical application still encounters some serious limitations that need to be addressed. These include loss of paracrine activity, rapid in vivo degradation of RNAs, poor in vivo cell viability, risk of carcinogenesis, viral infections, and off‐targeting.^18^, ^116^ Remarkably, long‐term controlled release of polynucleotides in vivo does not match in vitro conditions. The integrity of the vectors in vivo, both postinjection and in the long‐term, should also be investigated to avoid the leakage of polynucleotides. To date, the optimal kinetics and duration of polynucleotide release from biomaterial scaffolds have not been fully elucidated. Moreover, the survival and proliferation of stem cells during their expansion ex vivo is reported to be affected by oxidative stress.^117^ Other obstacles to this approach include electrostatic repulsion, which occurs when nucleic acids cross membranes with negative charge, and that delivery methods are short‐lived and do not provide sustained expression levels. Accordingly, numerous challenges must be considered, such as the optimal cell source, cultivation methods, vector type, promoter efficiency, dosage, and route of injection.^111^, ^112^, ^118^, ^119^ This section summarizes some well‐defined genetically cell mediation strategies employed in TE and regenerative medicine approaches.

#### 2.4.1 DNA

The interplay between gene delivery and regenerative medicine suggests the versatile therapeutic method. In recent years, polymeric scaffolds have been used as vehicles for gene delivery into specific cells to stimulate sustained transgene expression.^120^ Various cells are engineered to overexpress bioactive molecules that play crucial roles in tissue formation and repair.^121^, ^122^ Gonzalez‐Fernandez et al. have investigated the functionality of alginate hydrogels as supporters for encapsulating gene‐mediated MSCs with TGF‐β3 and BMP2 DNA plasmids; this complex caused rising secretion of sulfated glycosaminoglycan and collagen.^123^ In an in vivo study, glycosaminoglycan, containing osteogenic and angiogenic inducer genes (BMP‐2 and VEGF, respectively), was integrated into chitosan‐based scaffolds, resulting in total bone repair defects.^124^ In another investigation, Lackington et al. have focused on localized delivery of some neurotrophic factors via polyethyleneimine–pDNA nanoparticles incorporated into nerve guide conduit. Their results showed efficient delivery of NGF, GDNF, and c‐Jun transcription factor genes in both Schwann and neuronal cells. In vitro investigations presented enhancing regenerative cellular processes and neurite outgrowth.^125^

#### 2.4.2 RNA

Various types of RNA, including messenger RNA (mRNA), microRNAs (miRNAs), small interference RNAs (siRNAs), and small hairpin RNA (shRNA), can be used for orchestrating tissue regeneration signaling pathways through gene transcription modification, posttranscriptional gene silencing, and protein translation regulating in eukaryotic cells.^12^, ^126^ Due to better spatiotemporal control and safety concerns about direct gene delivery methods, RNA‐level gene expression modification provides an excellent opportunity for clinical applications.^127^, ^128^, ^129^, ^130^

Depending on the upregulation or downregulation of mRNA after tissue injury, various technologies such as miRNA‐based therapies and anti‐sense technologies can be used to manipulate and remodel the microenvironment of damaged tissue.^131^ Synthetic oligonucleotides, known as anti‐microRNAs, can specifically inhibit miRNA–mRNA interaction (Table 1).^132^, ^133^

**TABLE 1:** Some miRNA grafted scaffolds for tissue engineering purposes

Engineered tissue	Scaffold	Animal model (in vivo analysis)	Cell type (in vitro analysis)	miRNA	Molecular consequences	Result	References
Bone	PLGA/PLA	Mouse	Mouse osteoblasts	miRNA‐26a	Downregulation of Gsk‐3β and activation of Wnt/β‐catenin pathway	Upregulation of the osteoblastic activity of endogenous stem cells and bone repair	134
	PEG hydrogel	Rat	Human mesenchymal stem Cells	miRNA‐20a/siNoggin	Overexpression of BMP2	Promoting osteogenic differentiation	135
	Collagen	Rat	Rat bone marrow stem cells	miR‐148b	BMP signaling cascade	Activating osteogenesis and bone repair	136, 137
	Poly sebacoyl	Rat	Rat adipose‐derived stem cells	miR‐135	Repressing Hoxa2 expression/activating Runx2 pathway	Activating osteogenesis	138
Neural	PCL fibers	–	Rat oligodendroglial precursor cells	miR‐219/miR‐338	Inducing MBP expression	Promoting oligodendrocytes maturation	139
	PLLA/PCL	–	Trabecular meshwork mesenchymal stem cells	miR‐7	Overexpression of Nestin, GFAP, and MAP‐2	Enhancing glial and neural differentiate	140
Cardiovascular	PELCL/PCL/gelatin	Rabbit	Human umbilical vein endothelial cells	miR‐126	Downregulation of SPRED‐1 and PIK3R2	Acceleration of vascular endothelial cell proliferation	141
	PLGA/cRGD	–	Human umbilical vein ECs	miR‐132	Activation of Ras GAP‐1	Improving vessel‐forming	142
	Atelocollagen	Rat	Mouse myoblast	miR‐1/miR‐206/anti miR‐133	Overexpression of myogenic markers, downregulation of HDAC4 and MEF2C	Preventing fibrosis, promoting myotube differentiation	143

Target miRNAs that contribute to cell differentiation are usually detected by various screening methods.^144^ For example, blocking miR‐203 (sirtuin1 inhibitor) and miR‐449a (inhibitor of myeloid cell leukemia 1 factor) by their anti‐miR resulted in enhanced chondrogenesis in animal models.^145^, ^146^

In general, there are two ways to transform differentiated adult cells into other cell types: iPSC reprogramming and transdifferentiation, both of which require the expression of transcription factors. Synthetic mRNAs have shown great potential to facilitate cell engineering and reprogramming. They can be introduced into various somatic cells such as cardiomyocytes and translated directly into transcription factors by the cell's translation machinery.^127^, ^147^

Synthetic mRNAs can also be used for stem cell engineering because they can mediate safe, robust, and potent surface expression of molecules related to homing of stem cells. This is of great importance because when cultured in vitro, MSCs lack the potential for homing and cannot reach the target site, limiting their therapeutic efficiency.^127^, ^147^

With regard to spatial and temporal control of RNA delivery, injectable and in situ curing materials (e.g., hydrogels) are the most favorable candidates that allow minimally invasive approaches, reducing tissue damage, infection risks, and minimizing complications.^148^ Steinle et al. synthesized an injectable chitosan/alginate hydrogel loaded with HEK293 cells and synthetic mRNA to achieve exogenous protein synthesis in the target tissue.^149^ In another study, MSCs loaded with anti‐miR‐221 in fibrin and hyaluronan hydrogel were effectively converted into a chondrogenic line without using chondrogenic growth factors.^150^ Another group proposed an injectable thermoresponsive hydrogel to enhance local and sustained delivery of siRNA for tumor treatment.^151^ Balmayor et al. investigated the synergistic effects of fibrin gel/biphasic calcium phosphate granules and chemically modified BMP‐2 mRNA on stem cell osteogenesis. They showed that sustained release of BMP‐2 mRNA significantly promoted osteogenesis and mineral deposition.^148^ Neural tissue is an example of tissue type with limited regeneration capacity that may be a good candidate for RNA delivery for therapeutic purposes. Ma et al. demonstrated the role of miR‐29a in regulating ECM protein synthesis, neurite growth, and neural stem cell recruitment in the hippocampus.^152^ Upregulation of miR‐7 expression in human ocular MSCs seeded onto a nanofibrous PLLA/PCL scaffold resulted in differentiation of the cells into glial and neural progenitor cells.^140^ Although there are promising results on nucleic acid‐mediated scaffolds at TE, this field is still in its early stages. Synthetic mRNAs still present several disadvantages that limit their applicability, including the lack of a standardized and efficient synthesis protocol, low stability, and potential immunogenicity.^127^, ^147^ These limitations need to be addressed in the future to develop robust and efficient methods that can be translated into the clinical context.

### 2.5 Small molecules

Small molecules with low molecular weights (<1000 Da) include carbohydrates, lipids, amino acids, fatty acids, phenolic compounds, and alkaloids.^153^, ^154^ Considering their well‐identified physicochemical properties, good permeability, reproducible, low‐cost synthesis, and low immunogenicity reactions, small molecules can be used as pharmaceutical substances and cell differentiation factors.^17^, ^155^, ^156^ It is demonstrated that they can influence the endogenous stem cell lineage commitment, modulate specific intracellular processes and improve cell–cell and cell–scaffold interactions. Due to their function in cell manipulation, their application in TE is increasing. Some potential small molecules for use in TE are listed in Table 2.^10^, ^17^, ^155^, ^157^

**TABLE 2:** A list of small molecules for tissue engineering purposes

Engineered tissue	Small molecule	Target gene/pathway	Result	References
Bone	Bisphosphonates	Upregulation of BMP‐2, collagen I, osteocalcin, and alkaline phosphatase genes	Improving of osteoblast proliferation and inhibition of bone resorption	158, 159
	Doxycycline	Inhibiting of matrix metalloproteinase/neutralizing Dkk‐1/activation of Wnt signaling	Inhibiting osteoclast‐relating genes and bone resorption	160
	Simvastatin, Lovastatin, Rosuvastatin, Fluvastatin	Stimulating BMP/Smad pathway, activating BMP‐2 gene expression	Promoting neovascularization and osteogenesis	161, 162, 163
Cartilage	Compound‐6 (sulfonamide based)	Overexpression of aggrecan, activating MEK/ERK pathway	Promoting chondrogenesis	164
	Kartogenin	Overexpression of aggrecan and collagen II	Acceleration of MSCs chondrogenesis	165, 166
	Calcium silicate	Increasing glycosaminoglycan and collagen expression	Improving cell attachment and chondrogenesis	167
Skin	Pyrvinium	Inhibiting Wnt/β‐catenin signaling and activating casein kinase	Reducing myofibroblasts, fibrosis formation, and wound size	168
	XAV‐939	Inhibiting Wnt/β‐catenin and tankyrase	Reducing the number of myofibroblast and fibrosis formation	168
	Nitric oxide	Regulating of collagen production in fibroblasts	Accelerating re‐epithelialization, wound closure and hair follicle regeneration	169, 170
Neural	Isoxazole 9	Triggering of calcium‐activated pathway, CaMK phosphorylation and myocyte‐enhancer factor‐2	Enabling trans‐differentiation of astrocytes into neural cells/increasing neuron recruitment in the hippocampal dentate gyrus	171, 172
	Metphormin	Activating of aPKC‐CBP pathway	Stimulating neural stem cells recruitment and neurogenesis/improving spatial memory	173
	Forskolin	Activating of cAMP/PKA pathway and increasing intracellular cAMP	Promoting neurite outgrowth and axon polarization	174
Cardiovascular	Cardionogen	Inhibiting Wnt/β‐catenin signaling	Enhancing cardiogenesis	175
	Cyclosporin A	Inactivation of Calcineurin and NF/AT transcription factors	Increasing cardiomyogenesis and ESCs differentiation to cardiac cells	176
	CW209E	Over expression of cardiomyogenesis genes	Increasing number of beating embryoid bodies from human stem cells	177

Many small molecules specifically modulate intracellular signaling pathways for guided differentiation and determined stem cell fate. For example, Wnt/β‐catenin inhibitors including KY02111, IWR‐1, and XAV939, can robustly stimulate the differentiation of pluripotent stem cells to cardiomyocytes.^155^, ^156^, ^178^ Purmorphamine (an activator of the Sonic Hedgehog pathway) and DMH1 (an inhibitor of the BMP pathway) enable stem cells to differentiate into neuron‐like cells.^179^, ^180^

Small molecules are also commonly used for bone regeneration. These compounds can stimulate osteogenesis while effectively inactivating osteoclastogenesis.^181^ Simvastatin, lovastatin, and rosuvastatin are some members of the statin family that have been extensively studied for bone repair via affecting the BMP/Smad pathway. Tai et al. synthesized a PLGA/hydroxyapatite scaffold with simvastatin and found that it promoted neovascularization, cell growth, and osteogenesis.^161^, ^162^ In an in vivo experiment, lovastatin was encased in a polyurethane scaffold and injected into a bone defect model.^163^ Another study showed that purmorphamine and CW008, activators of the PKA/CREB pathway, promoted osteogenic differentiation of human MSCs.^182^, ^183^ Another study showed that a PLGA scaffold coated with FTY720 (an osteoinductive small molecule) significantly promoted the healing of cranial bone defects.^184^

### 2.6 Herbal extracts

In addition to synthetic agents, naturally derived small molecules, such as plant and bacterial secondary metabolites, could also effectively improve the biocompatibility of tissue‐engineered constructs.^185^, ^186^ Natural substances have shown great potential for bone TE because of their many advantages, such as their cost‐effectiveness and lack of side effects.^187^ Herbal extracts containing different types of phytochemicals (e.g., phenolic compounds, terpenes, phytohormones, and alkaloids) are attractive natural compounds that have achieved many investigations for healing purposes.^186^, ^188^ Some herbal extracts have been used in studies of stem cell proliferation and differentiation, including the aqueous extract of *Alpinia oxyphllae*,^163^ the extract of *Morinda citrifolia*, which induces osteogenesis differentiation,^189^, ^190^ and the extract of *Bacopa monnieri*, which promotes neurogenesis.^191^ Some small bioactive molecules such as gingerols and ginger essential oils (phenolic secondary metabolites in *Zingiber officinale*) have anti‐inflammatory, antibacterial, anticancer, hypoglycemic, and cardiotonic effects.^192^ In this context, Mohammadi et al. synthesized nanoliposomes containing ginger extract with pro‐angiogenic and antifungal effects and promoted wound healing process.^193^ In another study, an environmentally friendly method for developing ginger‐based nanofiber hydrogels as wound dressing with antibacterial activity was presented.^194^ Curcumin, the major secondary metabolite of *Curcuma longa* with antioxidant and anti‐inflammatory activity, enhances osteoblastic activity in addition to its antiosteoclastic effect.^195^ Bose et al. proposed novel 3D‐printed calcium phosphate scaffolds loaded with curcumin to improve the osteogenic potential of bone grafts. The results showed that liposomal release of curcumin could effectively promote osteoblast cell viability and proliferation in vitro and mineralized bone regeneration in vivo.^196^

Brahatheeswaran et al. prepared controlled‐release nanofibers loaded with curcumin, which resulted in enhanced adhesion and proliferation of fibroblasts.^197^, ^198^ Another example is garlic (*Allium sativum*), which contains various biologically functional secondary metabolites such as allicin (diallylthiosulfinate) that can be used for the treatment of wounds.^199^ Acemannan, a bioactive component of *Aloe vera*, has demonstrated excellent antibacterial, antioxidant, and immunomodulatory effects.^200^, ^201^ When used in combination with ADSCs, *Aloe vera* extracts incorporated in a hydrogel have been shown to significantly promote the healing of burn wounds.^202^

In recent decades, the use of herbal extracts in scaffolds has advanced modern TE technologies due to their bioactive, nontoxic, nonimmunogenic, and cost‐effective properties, as well as their mechanical strength.^186^, ^187^, ^188^ Therefore, herbal extracts have been used in approaches to promote tissue growth, to optimize bone and skin TE.^187^ Natural products in combination with polymer‐based scaffolds (e.g., chitosan and gelatin) can improve scaffold properties, including biodegradability and biocompatibility, enhance their physicochemical properties (e.g., wettability, thermal properties, morphology, porosity, and cell adhesion), modulate the response of cells, regulate immune responses, promote tissue formation at the site of injury or defect, and improve healing and regeneration.^186^, ^187^, ^188^ Remarkably, herbal extracts add anti‐inflammatory (inhibition of cytokine secretion and nitric oxide pathway), antioxidant, and antimicrobial (blocking DNA replication and metabolic pathways and inhibiting the synthesis of important proteins in bacteria) properties to engineered tissues to facilitate differentiation into the desired cell phenotype and tissue regeneration.^186^, ^188^ These properties are of great importance because infection, inflammation, sepsis, or oxidative stress are likely to compromise cell survival and, consequently, the efficiency of TE approaches.^188^ Alginate, starch, cellulose, and agar are plant polysaccharides that are also used for scaffold construction to make the biomedical use of scaffolds more efficient in terms of supporting cell growth, angiogenesis, and differentiation.^186^

These naturally derived scaffolds also provide a robust system for drug delivery by combining the advantages of scaffolds and plant extracts, allowing controlled release of the drug, with less toxicity and side effects.^187^ Some manufacturing technologies have been developed for drug delivery into biomaterials, such as freeze‐drying, solvent casting, 3D printing, electrospinning, and hydrogel formation, but there are still concerns about potential unsafe interactions at the implantation site and undesirable side effects.^187^, ^188^ Some potential herbal medicines for use in TE are listed in Table 3.

**TABLE 3:** A list of herbal medicine for tissue engineering purposes

Engineered tissue	Herbal medicine	Figure	Target gene/pathway	Result	References
Bone	*Alpinia oxyphllae*	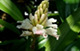	Suppression of RANKL/RANK pathway	Inhibition of osteoclast differentiation and bone destruction	163
	*Morinda citrifolia*	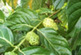	Activation of Wnt/βcatenin signaling and enhancement in ALP, Runx2, and OCN expression	Improvement of osteoblastic differentiation	189, 190
	*Curcuma longa*	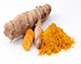	–	Osteoblastic activity enhancement	195, 196
	*Equisetum arvense*	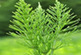	Enhancement in Runx2, Col I, and OPN expression	Increasing osteogenic differentiation, ALP activity, and mineralization content	203, 204
	*Spinacia oleracea*	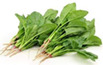	–	Prevention of loss of bone in osteoporosis	205
	*Cissus quadrangularis*	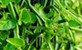	The activation of the early bone marker (ALP)	Invoking biomineralization and osteogenesis	206
Nerve	*Bacopa monnieri*	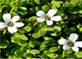	Activation of Akt and ERK1/2 signaling	Promotion of neural progenitor cells proliferation	191
Skin	*Zingiber officinale*	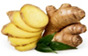	–	Enhancement in fibroblast cells proliferation	192, 193
	*Curcuma longa*	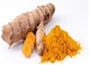	Inhibition of nuclear factor‐kappa B and effects on TGF‐β and MAPK pathways	Improvement of attachment and growth of fibroblast cells with free radical scavenging activity and reducing inflammation	197, 207, 208
	*Allium sativum*	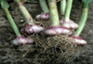	Affecting tight junctions and cytoskeleton distribution	High wound healing potential	199
	*Aloe vera*	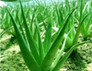	Increment in collagen synthesis and decrease in the collagen‐degrading MMP‐1 gene expression	Antibacterial, antioxidant, and immunomodulatory effects, improving wound healing	200, 201

### 2.7 Microbial‐derived compounds

Bacterial cells produce functional compounds that are suitable for many biomedical applications. An important advantage of microorganisms is that they can be genetically engineered to synthesize the desired compound for specific purposes. For microbial compounds to be used in TE, cost‐effectiveness, nontoxicity, and ease of processing are of paramount importance.^209^ Microbial polymers found in recent years include cellulose, alginic acid, agar, carrageenan, chitin, dextran, gellan gum, pullulan, and hyaluronic acid.^209^ Microbial cellulose fibers are mainly derived from a gram‐negative bacterium called *Acetobacter xylinum*. These fibers offer many advantages in terms of elasticity, safety, and high wettability, making them strong candidates for TE. Scaffolds for TE based on bacterial cellulose have been shown to improve chondrogenesis, osteogenesis, and stem cell differentiation compared to other bacterial components such as alginate and cellulose‐free scaffolds. In addition, the pellicle of bacterial cellulose could mimic the native collagen fibers of blood vessels, providing a suitable platform for muscle cell growth.^210^ Dextran, another component derived from bacteria, is used to develop scaffolds in combination with synthetic degradable polymers such as polyethylene glycol and polylactic acid. Scaffolds containing dextran can support proliferation and adhesion of cells and degrade in vivo. Moreover, these scaffolds showed an increase in Young's modulus by combining with nanohydroxyapatite.^210^

Poly‐gamma‐glutamic acid and polyhydroxyalkanoates are other bacteria‐derived polymers of great interest in scaffold development and drug delivery because of their superior properties in homeostasis, cell adhesion, proliferation, and survival.^209^ Microbial secondary metabolites, such as antibiotics, growth hormones, and antitumor agents, have great potential for medical applications. For example, in one study, incorporating the fluoroquinolone antibiotic levofloxacin into the electrospun hybrid scaffold promoted cellular activities and reduced potential postoperative inflammation and infection.^211^ The sustained release of the polyketide antibiotic tetracycline from the core–mantle fibers could inhibit microbial infections.^212^ In another study, Ramalingam et al. investigated the synergistic effects of the tetracycline antibiotic minocin and *G*. *sylvestre* extracts on re‐epithelialization and collagen organization during wound healing by a core–shell mat.^213^ In addition, biopolymers derived from bacteria, polysaccharides (such as alginate, dextran, gellan gum), polyesters such as polyhydroxyalkanoates, polyamides (such as poly(γ‐glutamic acid), poly(ε‐l‐lysine)), and inorganic polyanhydrides (such as polyphosphates) are biocompatible and biodegradable candidates for potential TE applications.^214^, ^215^

In addition to microbial compounds, probiotics, living organisms that benefit host health, have also been found to hold promise for TE.^216^ Probiotics are a group of living microorganisms that are effective in sufficient quantities for cancer, inflammatory diseases, and gastrointestinal infections. However, probiotics are sensitive to high temperatures, acidic conditions, and high oxygen levels, so encapsulation of probiotics serves to protect the bacteria and maintain their metabolic activity. The function of the encapsulation carrier is to provide a suitable microenvironment in which the bacteria can survive during processing and storage and be released at the desired site in the gastrointestinal tract. Probiotics can be used for wound healing, protection from harmful radiation, and enhancement of innate immunity.^217^ In one study, the effective role of *E*. *mundtii* against infections was investigated. In vivo results showed a synergistic role of probiotic matrix and nanostructure to improve wound healing after burns.^218^ Scientists studying the role of scaffolds functionalized with probiotics have shown that they have an anti‐inflammatory effect and thus increase the healing rate.^216^

## 3 BIOPHYSICAL FACTORS

Although biochemical induction factors are widely used to create ECM‐like microenvironments, nowadays, growing studies report significant contributions of biophysical inducers in TE. There are numerous reports about the effects of heat,^219^, ^220^, ^221^ ultrasound waves,^222^ magnetic fields,^223^ electrical,^224^, ^225^, ^226^ mechanical,^227^, ^228^, ^229^ and light stimulation on cellular phenotype and function.^230^, ^231^ Furthermore, different topographical aspects of the matrix, such as surface stiffness, elasticity, and porosity, could be considered as inductive factors to modify cell fate determination. Various methods are used to functionalize the structure of the scaffold to induce favorable cell behavior.^19^, ^232^

The selection of appropriate induction factors must take into account the structural and functional features of the target tissue. Due to the physiological nature of electrical stimulation in the normal function of some tissues such as nerves, bone, muscles, and heart, electrical stimulation and electroconductive polymers have been remarkable research topics in recent decades.^226^ Based on the electromechanical properties of cardiac tissue, the combination of electro‐conductive materials with suitable mechanical properties and electrical stimulation could be considered a promising tool in cardiac TE.^11^, ^233^

**FIGURE 2:**
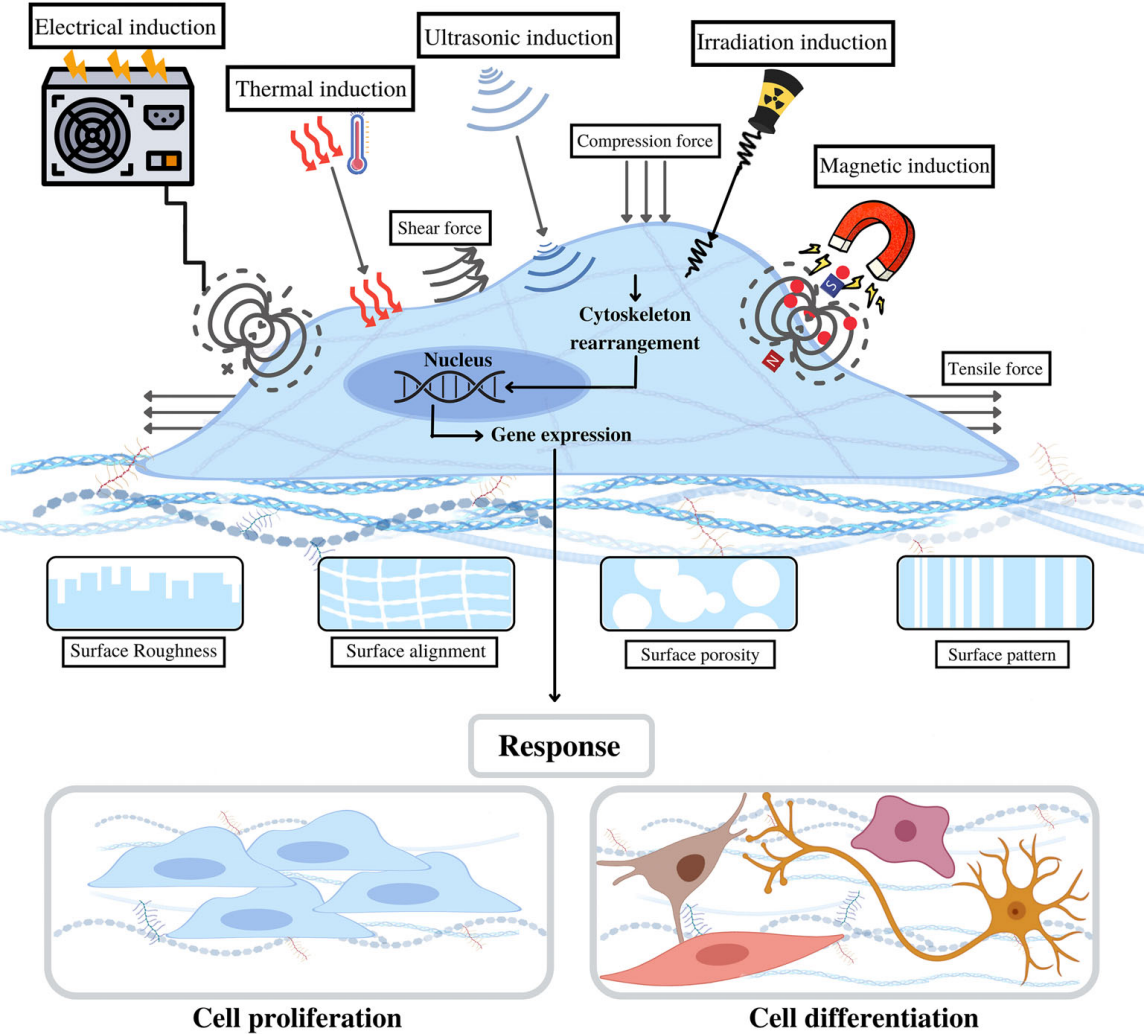
Biophysical stimulating factors involved in manipulating cell fate decisions in tissue engineering. Various biophysical stimuli can be applied to cells to obtain the most preferable cell behavior and characteristics; magnetic, thermal, ultrasound, irradiation, electric, and mechanical (shear stress, tensile, fluid flow, hydrostatic pressure, and compression forces) induction are among these stimuli. Moreover, various surface topographies give rise to different cells responses. Stimulatory factors upon affecting sensitive receptors of the cell surface, contribute to rearrangement and reorientation of cell cytoskeletons. Once in the nucleus, the signals generated by cytoskeleton rearrangement leads to alternation in the gene expression which determines the cell behavior consequently

Overlaps between biophysical and biochemical signals regulate various aspects of a cell life cycle. Biophysical factors regulate various intracellular signaling pathways by affecting sensitive receptors on the cell surface. However, the molecular mechanisms of physical cues are not fully understood. Due to the importance of the physical and mechanical properties of tissues to their natural function, the use of biophysical inducers has recently been emphasized for more effective TE. These biophysical stimuli may provide cost‐effective, safe, and nondestructive tools for regenerative medicine. In this section, the effects of various physical and mechanical stimuli on cell fate determination, cell function, and cell–cell integrity are presented and discussed in the following subsections and summarized in Figure 2.

### 3.1 Surface topography

**FIGURE 3:**
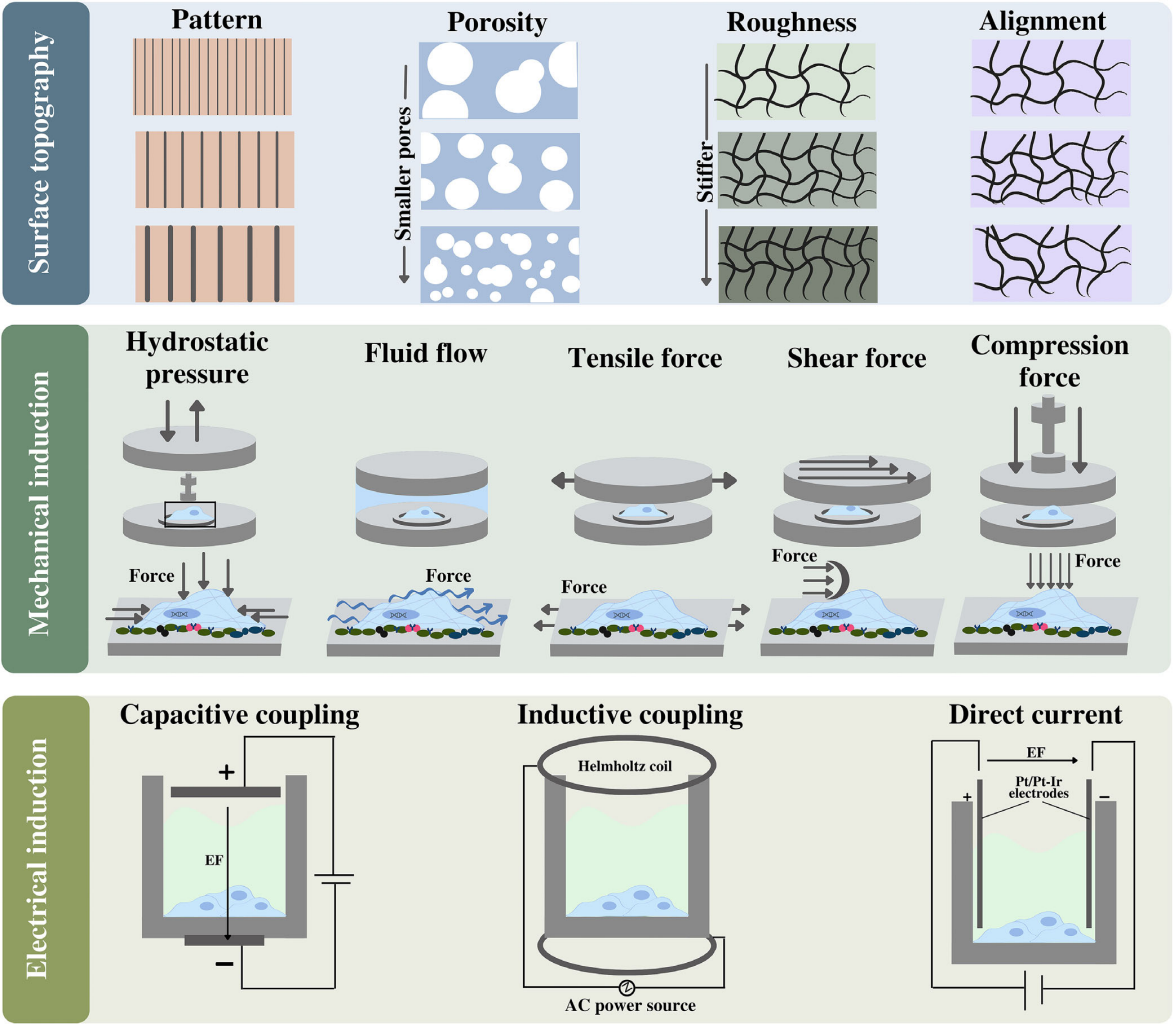
Various aspects of surface topography, Different types of mechanical stresses, and electrical stimulations in tissue engineering. (Top) Different surface topographic textures have different impacts on cell activities and responses. Four different aspects of topography demonstrated in the figure, are surface patterns, roughness, alignment, and pore size. (Middle) Mechanical forces in cell niches such as compression, shear, fluid flow, hydrostatic pressure, and tensile can be sensed by cells through diverse mechanisms. The direction of arrows illustrates the direction of mechanical force. (Below) Electrical stimulation along with parameters involved in electric current play crucial roles in the cell behavior. Three methods to induce electric current are direct coupling, capacitive coupling, and inductive coupling

The topographic properties of a surface encompass three general concepts: roughness, pattern, and porosity (Figure 3).^234^ Scientists have taken advantage of these properties to efficiently engineer various tissue types by harnessing the effect of surfaces in controlling cell behavior, including cell proliferation, migration, adhesion, and differentiation.^234^, ^235^, ^236^, ^237^ Therefore, elucidating how surface features control cell fate is key to developing optimal implants for TE. Interestingly, these topographic features have been observed to act synergistically with soluble factor‐mediated signaling.^235^ The methods used to engineer the surface topography generally fall into two groups: creating protrusions and/or depressions on a micro/nanoscale, or altering the mechanical properties of the material in a predefined pattern. These patterns can be arranged isotropically (e.g., columns, pits, or tubes) or anisotropically (e.g., grooves and ridges). Micro/nanoscale patterns have been shown to affect stem cell attachment by altering cytoskeletal orientation and intracellular focal adhesions that can modulate intracellular signaling and mechanotransduction pathways.^7^, ^234^, ^236^, ^238^, ^239^, ^240^ Surface patterning allows the regulation of biomaterial–host tissue interactions, enabling their application in the development of various tissues.^234^, ^235^ Lee et al. synthesized nanostructured ridge/groove pattern arrays to study the effects of controlling topological dimensions and orientations on neurogenesis of hESCs.^236^ Interestingly, neural progenitor cells may respond differently to the physical presence of cues, as anisotropic topographies have been shown to significantly facilitate neural differentiation, whereas isotropic topographies promote glial differentiation.^236^ Lee et al. synthesized the nanostructured ridge/groove pattern arrays to study the effect of controlling topological dimensions and alignments on hESCs neurogenesis.^241^ Interestingly, neural progenitor cells may respond differently to the physical presence of cues, as anisotropic topographies have been shown to significantly facilitate neural differentiation, whereas isotropic topographies promote glial differentiation.^242^ Surface patterns are also critical for bone tissue homeostasis, as they regulate osteoblasts in maintaining their characteristic function. The major signaling mechanism that appears to be involved in the transmission of microtopographic information to osteoblasts is the RhoA/ROCK signaling pathway.^243^

The other aspect of surface topography, porosity, plays an essential role in cell functionality, nutrient transport, cell fate determination, cell migration, adherence, and growth.^234^ In this sense, scaffolds for bone tissue with appropriate porosity led to osteogenesis, angiogenesis, and enhanced oxygen transfer to cells.^234^ Pore size, shape, uniformity, and the degree of interconnectivity between pores, are some of the critical elements to consider. In addition, the influence of pore size seems to depend on the cell type and biomaterial of the matrix.^7^, ^234^, ^244^ Zhang et al. indicated that pore size alters the proliferation rate of MSCs^245^ and in another study, culture of MSCs in collagen/hyaluronic acid scaffolds with three different pore sizes showed the highest expression of cartilage marker genes and deposition of cartilage‐like matrix in the substrate with the largest pore size.^246^ Accordingly, increasing porosity has a positive effect on nutrient diffusion and better removal of cellular debris, leading to better proliferation rate and osteogenesis of MSCs in vivo.^247^ Brennan et al. fabricated a fibrous scaffold architecture to investigate the effect of different scaffold pore sizes (100, 200, and 300 μm) on osteogenesis of MSCs. Their results showed that cell infiltration into these porous structures was better than into high‐packed random fibrous scaffolds. In addition, a matrix with a pore size of 100 μm significantly promoted cell infiltration, spreading, and mineralization.^248^

The other topographic parameter, roughness, is defined as the extent of protrusions and depressions on the surface of the biomaterial Chen et al. investigated the effects of fiber surface roughness by changing the moisture ratio during electrospinning. The results suggest that hMSCs seeded on the roughest surface produced more osteogenic markers including osteopontin, BMPs, and Runx2. Yang et al. prepared hyaluronic acid matrices with a different average roughness (ranging from 0.2 to 1.65 μm) and average spacing between tips (ranging from 89.7 to 18.6 μm) to study the osteogenic differentiation of MSCs. Their results showed that matrices with a roughness of 0.7–1.0 μm and peak spacing of 53.9–39.3 μm had better osteogenesis potential.^249^ Interestingly, it appears that cell behavior is controlled by the combined contribution of the micro and nano‐roughness of the substrate.^240^

### 3.2 Mechanical cues

Cells can sense the mechanical properties of their environment through diverse mechanisms including mechanoreceptors, focal adhesions, actin cytoskeleton, and cell–cell interactions. Matrix–cell adhesion mechanosensors connect the ECM and the actin cytoskeleton by transmembrane connections. Inside the cells, mechanical stimuli are converted to arrays of biochemical cascades transforming information to nuclei, leading to reorientation, and reorganization of the cell cytoskeleton.^229^ These force‐induced modifications influence gene expression and change cell protein profiling. Rho/ROCK, Rac/MAPK, Src/FAK, PI3K/Akt, and Wnt/β‐catenin are some of the key mechanotransduction pathways that transform mechanical information into nuclei and reorganize cytoskeletal network.^5^, ^250^ So, the mechanical properties of cell niches significantly impact cell quiescence, division, and differentiation (Figure 3). During embryonic development, mechanical forces are involved in tissue formation, and organogenesis by affecting cell migration, fate determination, and matrix composition reorganizing. In response to mechanical stress, cells begin to produce more tissue‐specific ECM components.^251^ In a study, short bursts of compression to human embryonic mesodermal progenitor cells led to increased mineral deposition and osteogenic differentiation.^252^

Accordingly, forces such as fluid shear stresses, hydrostatic compression, mechanical tension, and bending take part to participate in cell fate determination.^253^, ^254^ The major influence of matrix stiffness on cell differentiation and migration is also reported.^255^, ^256^ The stiffness of the matrix regulates the type and the intensity of cell–substrate forces. On a stiff substrate, cells generate a large force at the focal adhesion, that affects the lineage specification and commitment.^257^ A study showed that elastic environments favored differentiation of MSC into adipocytes, while osteogenesis was enhanced on stiffer substrates.^255^ Sun et al. demonstrated that MSCs cultured on soft hydrogels, significantly increased the activation of b1‐integrin compared to stiffer matrix, playing an important role in modulating differentiation and self‐renewal of MSCs.^258^

A variety of mechanical stimuli can be applied to cells using flexible culture substrates as well as scaffolds or bioreactors with suitable mechanical properties.^87^, ^259^ Axial, biaxial and cyclic static stresses have been used to induce cell proliferation.^251^ Studies have shown that cyclic mechanical strain is determinant in the control of the assembly of cells grown on flexible substrates.^260^, ^261^ In an in vitro study, Pennisi et al. provided evidence on the role of uniaxial cyclic tensile strain (CTS) in the differentiation of skeletal myocytes. They found that applying CTS on cells resulted in a highly aligned array of cross‐striated fibers to the direction of strain and accelerated maturation of myoblasts.^262^ Another study reported that stimulated MSCs with cyclic stretching represented increased proliferation and cardiomyogenic differentiation.^263^ In continuation, cyclic stretching showed a negative effect on cell proliferation but a positive effect on osteogenesis.^264^ Kong et al. developed a micro‐device for applying cyclic compressions to cardiac fibroblasts (CFs) encapsulated in hydrogels resulting in the strain–response correlation of mechanical stimulation in CFs phenotypic remodeling.^227^ In addition, stress relaxation of the substrate can be an important mechanical parameter that influences on cell behavior.^265^ Seeding of murine MSC in a tunable stress relaxation hydrogel revealed that the proliferation and spreading rate of MSCs on the fast stress relaxation substrate were extremely higher than the low relaxation hydrogel. As well, the rapid stress relaxation matrix increased the bone formation and osteogenesis of MSCs.^266^ In another study, the simultaneous investigation of stiffness and topography of different polyesters introduced the nanopillar structures with lower stiffness with better conditions for chondrogenesis than the others.^267^

### 3.3 Electrical cues

Electrical currents generated by the cells and ECM components during development, growth, or repair are known as bioelectric phenomena. Cells display a resting membrane potential which is mainly due to the differential concentration of ions across the cytoplasmic membrane. At rest, ion channels help maintain the electrochemical gradient of ions across the membrane. Activation of ion channels can lead to changes in the membrane potential, which is typically associated with a functional response in the cells. Many biological processes such as neuron synapses, muscle contraction, bone formation, and wound healing in human body are modulated by electrical fields.^233^, ^268^ Electrical stimulation has been used in combination with various inductive factors such as growth factors,^269^ small molecules,^11^ electro‐conductive scaffolds,^270^ and even mechanical stimuli for efficient TE (Figure 3).^271^

Usually, one of the three following electrical stimulation methods is applied: direct, capacitive, or inductive coupling. Direct coupling is limited by the fact that stimulation electrodes can produce cytotoxic by‐products and alter the pH. Capacitive coupling produces a uniform electric field by two opposite electrodes, without the need for electroconductive scaffolds. Inductive coupling can be produced by an electromagnetic field created by an external coil. In general, studies have shown that parameters such as frequency, intensity, and duration of the stimulation play an important role in cell behavior.^268^, ^272^

Due to the natural function of electrical pulses in cardiac and neural tissues as well as osteogenesis‐related processes, the use of conductive scaffolds and electrical stimulation in these areas has been very effective.^11^, ^233^ A study has shown that using a high‐intensity electrical field stimulation with conductive scaffolds provided an excellent stimulant to improve wound healing in tissue injuries due to its effect on cell membrane permeability.^224^ Another study demonstrated that biphasic square pulses stimulated proliferation and enhanced differentiation of ADSCs to osteoblast.^268^ Abedi et al. showed that the combined application of conductive scaffold and electrical stimulation significantly increased in the expression of cardiac marker genes in cardiac progenitor cells.^11^ Another study in neural TE reported that synergic use of electrical stimulation and conductive scaffolds caused the upregulation of neural marker genes in stem cells.^233^ Zhao et al. presented that the in vivo growth and regeneration of axons were effectively increased on conductive polypyrrole/silk fibroin scaffolds under electrical stimulation, while the signal transduction pathway of MAPKs was activated.^273^ Bjorninen et al. presented a 3D, reusable and cost‐effective electrical stimulation device to differentiate ADSCs to SMCs on polytrimethylene carbonate scaffolds coated with polypyrrole. The use of biphasic electric current with a pulse width of 1 ms increased the number of living cells compared to unstimulated ADSCs and supported the expression of SMCs markers.^274^

### 3.4 Low‐frequency magnetic fields

Most molecular materials are diamagnetic, a weak form of magnetism created by a magnetic field as a result of the change in the orbital motion of the electrons. The force that arises from the motion of electrons at the molecular level is very small, so the effect of diamagnetism can be increased by applying magnetic fields.^275^ Studies on fibroblasts and osteoblasts seeded on magnetic polymer nanofibers have shown improvement in cell alignment and proliferation. Another study demonstrated increased chondrogenesis of MSCs by stimulating an external magnetic field on hydrogel scaffolds.^276^ Pulsed electromagnetic fields (PEMFs) can accelerate fracture healing and osteogenesis in vivo, probably due to the activation of the biological cascade. Improvement of cytosolic Ca^2+^ and calmodulin activation is one of the impacts of PEMF exposure, which enhances the osteogenesis process. Efforts to investigate the differences between static magnetic fields and PEMFs have shown that using static magnetic fields with moderate intensity have more beneficial results in metabolic disorders treatment, while in musculoskeletal and neurological disorders, PEMFs have more favorable effects. It is noteworthy to mention that stimulation intensity, frequency, and duration of the magnetic field are important parameters to consider.^277^, ^278^ Özgün et al. investigated the role of N‐methyl‐d‐aspartate receptors in neural differentiation by exposing differentiated human neural progenitor cells under extremely low‐frequency magnetic field; an increase in the levels of neuronal markers expression and neurites outgrowth was observed.^279^

### 3.5 Ultrasonic fields

The ultrasound wave is a high‐frequency mechanical wave that is widely used for medical applications due to its noninvasive, deep tissue penetration and remote controllability. This nondestructive stimulation approach can be passed through human tissues as a biophysical signal in a controlled and systematic manner. It can also stimulate complex biochemical events in cells. Small‐scale tissue models are used to improve our understanding of biological systems. In this regard, ultrasound has the capability to arrange cells in favorable patterns with micrometer‐sized features.^280^ Ultrasound waves alter cellular metabolism by applying energy to the cellular cytoskeleton.^222^ Recently, 2D and 3D acoustic cell patterns have been considered to stimulate biological processes including neuron conduction, neural differentiation, cardiomyocyte contractility, and angiogenesis.^281^ Studies of low‐intensity pulsed ultrasound stimulation (LIPUS) to induce mechanical strain and, consequently, biochemical changes have shown that LIPUS stimulation can increase osteoblasts activity and bone differentiation of precursor stem cells.^282^ Also, LIPUS could regulate the cell cycle by activating ERKs/MAPKs pathways and also affecting fibroblast proliferation.^283^ Cheng et al. for the first time presented a simple, cost‐effective, reproducible ultrasound standing waves (USWs) system to repair the peripheral nerve. USWs radiation did not have any adverse effect on cell viability, differentiation, and proliferation. Axonal growth of differentiated PC12 cells by USWs had a higher directional uniformity than the control group.^284^ USWs have been utilized in tissue explant cultures, drug delivery, tissue modeling, immunology, and cancer studies. USW particle manipulation provides aggregation of cells. Depending on actuation schemes, the pressure nodes defined by the USW are stable and can be both static and dynamic, while usually the cells are trapped in the mentioned nodes.^280^ The components of the ultrasonic device include a signal generator and an ultrasonic transducer that can produce constant ultrasonic frequencies depending on the power and excitation time.^285^

### 3.6 Thermal energy

Chemical reactions in cellular processes are temperature sensitive, both for protein activation or inactivation and for subsequent signaling pathways. Hyperthermia, in general, is used for cancer treatment due to its deleterious effects on cells,^286^, ^287^ whereas moderate temperature elevations could find application in TE. Ma et al. developed a new temperature‐sensitive hydroxyapatite/graphene oxide/CS platform to kill osteosarcoma cells at a temperature of 48°C by near‐infrared radiation.^288^

Several studies described the beneficial effects of controlled hyperthermic therapy and mild heat stress on myogenic and osteogenic differentiation. For instance, incubation of cultured myoblasts at 39°C showed an increase in myotube diameter (skeletal muscle hypertrophy).^220^ Another study examined the effect on myotubes of mild temperature increments (within the physiological range), produced by near‐infrared radiation using gold nanoshells, The result was a significant positive and protective effect of heating on C2C12 myotube contractility.^289^ An in vivo study reported elevated temperature from 1.5°C to 3°C more than body temperature motivated bone growth.^290^ Tong et al. investigated the effect of low intensity and periodic near‐infrared radiation on osteogenic differentiation of seeding cells on black phosphorus nanosheets and PLGA substrate. They found that moderate heat stimulation promoted heat shock protein expression following osteogenesis and bone healing acceleration in vitro and in vivo.^291^ Sanchez‐Casanova et al. explored the influence of photo‐induced hyperthermia on the liberation of BMP‐2 from near‐infrared radiation‐responsive hydrogels for bone TE, which led to the controlled release of BMP‐2 for enhanced performance bone formation and mineralization in vivo.^292^

### 3.7 Nonionizing, nonthermal light treatment

Stimulation of cells by nonionizing radiation leads to the absorption of energy by various cellular mechanisms. Recently, light radiation emanating from light‐emitting diodes (LEDs), has been considered as a physical agent with an extraordinary ability to modulate the proliferation and differentiation of various cell types. The term photobiomodulation, as a nonthermal method, refers to the biological effects that occur after electromagnetic waves in the visible and infrared range interact with molecules and cells. Photobiomodulation has the ability to turn on or off signaling mechanisms related to cell growth and metabolic activities by changing the parameters of wavelength and energy density.^230^, ^293^ It was reported that photobiomodulation effectively prevented or mitigated complications of radiotherapy by preconditioning the cells to reduce inflammation and promote tissue healing.^294^ LED light, by stimulating the mitochondrial ATP synthesis, has been shown to significantly modify cellular metabolism. This method has been used to promote the proliferation of osteoblasts and accelerate bone repair.^295^

Photobiomodulation also enhances the rate of tissue healing by increasing the differentiation of sensitive and precursor stem cells.^296^ Ruan et al. showed that high‐intensity red LED irradiations positively affected the osteogenesis of hMSCs through activation of the Wnt/β‐catenin signaling pathway. However, it did not have a significant effect on cell proliferation after long‐term culture.^297^ In another investigation, irradiation with 660 nm wavelength on astrocytes enhanced nestin and aldh1L1 expression and reduced Oct4 and GFAP co‐expression simultaneously.^298^ Mokoena et al. used photobiomodulation with a wavelength of 660 nm to trans‐differentiate fibroblasts to myofibroblasts for diabetic wound healing. The results confirmed that wound closure occurred even without proper activation of the TGF‐β/Smad pathway, so that a different signaling pathway was activated during the transdifferentiation of fibroblasts to myofibroblasts.^299^ In another study, Funch et al. examined the effect of 660 nm wavelength on the trans‐differentiation of ADSCs to fibroblasts and chondrocytes. Their findings suggested the potential use of using light stimulation at this wavelength for the treatment of temporomandibular disorder.^300^

Noninvasive laser radiation can modulate cellular behavior and tissue repair by creating a photomodulator effect on cells and tissues.^230^, ^231^ One recent study suggested applying a noninvasive photobiomodulation therapy in the range of 820–840 nm wavelength to accelerate bone regeneration processes combined with the alloplastic ceramic and fibrin biopolymer scaffolds. After 42 days, progressive incensement in bone repair revealed the potential application of this designed platform for bone TE.^301^

## 4 FUNCTIONAL SYNERGY BETWEEN DIFFERENT STIMULI

Since cell adhesion, proliferation, and differentiation are multifactorial mechanisms that are triggered or controlled by various biochemical and biophysical stimuli, recent studies have focused on the functional synergy between these different stimuli to establish efficient TE approaches for clinical application. For instance, a study reported the ability of an electro‐conductive scaffold integrated with BMP‐4 plasmid for efficient bone regeneration.^302^ Zhang et al. investigated the synergistic effects of topographical cue of the scaffold, electrical stimulation, and neural growth factor on PC12 cells simultaneously. The results demonstrated that sustained release of NGF from core–shell nanofibrous scaffold under electrical stimulation considerably improved cell proliferation and neurite outgrowth.^303^ In another research, the synergistic effects of electrical stimulation and small molecules (including IWP2, CHIR99021, purmorphamine, and SB431542) resulted in the overexpression of main cardiac marker genes that was suggested as a suitable cardiac repair platform.^11^ Another investigation has focused on the synergistic effects of chemical and mechanical stimulations on cartilage regeneration. After preconditioning of MSCs in chondrogenic differentiation media, cells were loaded in a 3D hydrogel with subsequent dynamic compressive loading. The implantation of this construct in an osteochondral defect of a rat model showed great enhancement in cartilage repair.^304^ Zhang et al. designed a multifunctional hollow‐pipe structure to control ions release for vascularized bone TE. Due to the remarkable synergistic effects of the structure and bioactive ionic composition of the Ca_7_MgSi_4_O_16_ scaffold, the angiogenesis and bone regeneration rate were significantly promoted. Their findings revealed this multifunctional platform not only improved the infiltration of host blood vessels into the hollow struts but also showed great opportunity for cells and growth factors delivery.^305^ Synthesized thermally induced hydrogel/curcumin by Pham et al. showed promoted fibroblast adhesion and faster wound closure process.^306^ Another study demonstrated that the combined application of TGF‐β1 and cyclic stretching could enhance tendon healing.^307^ Taken together, these studies highlight the importance of synergism between various biochemical and biophysical inducers for more effective mimicking of the natural niche. Investigation of synergism between different stimulators and optimized exploitation of the interactions could lead to more efficient differentiation protocols for TE.^226^

## 5 CLINICAL TRIALS AND CHALLENGES TO TRANSLATION

The first clinical attempts to use tissue engineering took place during 1980s to repair skin and articular, and cartilaginous surfaces in human and animal specimens.^308^ Data analysis of tissue engineering and regenerative medicine patents by machine learning has shown that most patents focus on the integration of stem cells into scaffolds based on natural polymers (e.g., chitosan, collagen).^309^ The development of a commercial tissue‐engineered construct generally requires the performance of multiple preclinical and clinical studies. In addition, regulatory agencies such as the Food and Drug Administration (FDA) have precise requirements for clinical efficacy and safety studies.^310^ The use of tissue engineering platforms for clinical translation remains challenging in terms of producing sufficient tissue engineering tissues in compliance with good manufacturing practices (GMP). Despite significant efforts to advance tissue engineering and regenerative medicine, few products have been successfully translated into clinical practice; of those, only about 14% have been able to obtain FDA approval. This failure to implement tissue engineering and regenerative medicine has several causes, for example, lack of funding, financing, and clinical approval.^311^

Wound healing scaffolds, nerve and small vessel grafts are tissue engineering based products currently on the market; however, they have not had significant regulatory success.^312^ Among the therapies that have paved their way to the clinics, autologous cell‐based therapies (e.g., Carticel, autologous chondrocytes for cartilage repair) and decellularized scaffolds have demonstrated the most promising outcomes.^313^, ^314^ However, the need for one‐to‐one transfer, adequate cell density, requirements for sophisticated clinical bioreactors, and the sterilization process during scaffold manufacture pose a significant problem for clinical implementation.^315^ Some clinical trials of cell‐based tissue‐engineered products are mentioned in Table 4.

**TABLE 4:** Clinical trials of cell‐based tissue‐engineered products

NTC number	Phase	Indication	Intervention	Cell type	Outcome	References
NCT02698813	I	Scar, senescence wrinkles	Intradermal injection of HA and UC‐MSCs	UC‐MSCs	Improvement of wrinkles, acne, and pitting scar	Web ref: 1
NCT01981330	I	Vocal fold scarring and hoarseness	Injection of BM‐MSCs with a carrier hyaluronan gel	BM‐MSCs	Healing scarred vocal folds	Web ref: 2
NCT02123368	I/II	Osteoarthritis	IA injection of BM‐MSCs and HA	BM‐MSCs	Improvement of the osteoarthritis	Web ref: 3
NCT03384433	I/II	Acute ischemic stroke	Intraparenchymal administration of allogenic MSC‐EVs with miR‐124	Allogenic MSC‐EVs	Able to look after own affairs without assistance	Web ref: 4
NCT04173650	II	Dystrophic Epidermolysis Bullosa	Allogenic locally injection BMMSC‐EVs	BMMSC‐EVs	Not published	Web ref: 5
NCT03857841	I	Bronchopulmonary dysplasia	Intravenous infusion of BMMSC‐EVs	BMMSC‐EVs	Improvement of the disease	Web ref: 6

*Note*: Web References—1. https://clinicaltrials.gov/ct2/show/NCT02698813; 2. https://clinicaltrials.gov/ct2/show/NCT01981330; 3. https://clinicaltrials.gov/ct2/show/NCT02776943; 4. https://clinicaltrials.gov/ct2/show/NCT03384433; 5. https://clinicaltrials.gov/ct2/show/NCT04173650; 6. https://clinicaltrials.gov/ct2/show/NCT03857841.

Notably, numerous scaffolds have been developed clinically for the treatment of bone, skin, cartilage, and bladder injuries.^315^, ^316^ A 3D‐bioprinted cell‐free poly‐ε‐caprolactone implant has received FDA approval as a bioresorbable airway splint for use in lower airway damage.^317^ Allogeneic scaffolds from the iliac vein, incorporating autologous smooth muscle cells and endothelial cells, are also used in clinics to treat extrahepatic portal vein obstruction.^6^ In 2002, the incorporation of BMP‐2 growth factor into a collagen sponge to support bone regeneration in the INFUSE bone graft was approved by the FDA.^318^ Another FDA‐approved collagen‐based scaffold named Osigraft® (Stryker Biotech) contains recombinant BMP‐7 used to treat tibia fractures.^319^ The Integra® Template, made of collagen, glycosaminoglycans, and polysiloxane, is another FDA‐approved device for burn treatment and wound healing that uses ECM‐derived components for clinical purposes.^320^ Dermagraft® is a cryopreserved dermal substitute developed by culturing neonatal fibroblasts onto a bioabsorbable polyglactin scaffold that has been approved for marketing in the US for diabetic foot ulcer treatment.^321^

Despite the enormous potential of using biophysical stimuli in TE, little research has been done on the use of these triggers at the clinical level. Scientists' priority on product efficacy and lack of attention to regulations for a product's clinical and commercial applicability is a major reason why tissue engineering‐based products fail to meet clinical criteria. For example, bioprinted 3D tissues lack the necessary regulations for clinical trials.^309^ These regulations are factors such as administration time, price, and ethics. Therefore, it is imperative that scientists and investigators follow clinical criteria regulations and apply a bedside to bench approach and back again to overcome the barriers to clinical acceptance of tissue engineering‐based therapy and increase its clinical impact.^311^

Overall, barriers to the introduction of tissue engineering‐based therapies and products into clinics and the marketplace fall into three categories: technical challenges (e.g., optimal cell source, immune rejection, appropriate microenvironment, proper vascularization, and optimized delivery), manufacturing challenges (e.g., scale‐up, timelines, shelf life, and price), and regulatory challenges (e.g., low percentage of approvals and guidelines).^309^ To address these challenges, the tissue engineering community has undertaken numerous efforts in three main areas, including the differentiation of iPSCs, the development of biomaterials, morphogens, and growth factors, and the use of decellularized ECM and bioprinting to create suitable tissue structures.^309^

A group of researchers argues that many of the efforts in the field of tissue engineering will not have clinical applications because of the lack of FDA approval for biomaterials and their components. Requirements for a commercial biomaterials template include biodegradability of biomaterials with appropriate kinetics, ability to permanently adapt to the changing environment of the body, transmission of molecular signals to target cells by biomaterials, appropriate mechanical properties by adapting to the target tissue, ability to inject into target tissues on demand, optimization of the transfer of nutrients and biological molecules and gases to cells, ability to facilitate the development of blood vessels and neural networks, adaptation to modern manufacturing techniques such as 3D printing, nontoxicity and immunogenicity in the body.^322^ On the other hand, some reports attribute the limited use of TE technology in the clinical field to the economic conditions in the industry and the high cost of TE research, which makes many investors reluctant to invest.^311^, ^323^

With the introduction of modern technologies in tissue engineering, such as microfluidics and advanced scaffold fabrication methods such as 3D printing, hopes for clinical application have increased. However, this process is still slow, and obstacles such as the lack of scalability of biomaterials for use in clinical cases, the lack of a unified monitoring protocol for scaffold fabrication processes and supply of materials and cell banks, and the lack of clear instructions for the production of standard biomaterials that do not elicit an immune response are the main issues to be overcome. Also, the inefficiency of sensitive and expensive laboratory solutions for physicians and surgeons, who are looking more for solutions with greater reliability, ease of use, and, at the same time, lower costs, are another obstacle to the nonentry of tissue engineering achievements into commercialization and have led to the fact that classical and old methods are more welcomed by surgeons.^323^

Despite the above limitations, the value of tissue engineering techniques in medicine will not diminish, and with technological progress, tissue engineering has the opportunity to improve and develop for therapeutic purposes and organ replacement.

## 6 SUMMARY, CONCLUSIONS, AND PERSPECTIVES

Mimicking the natural cell environment, both as a physical supporter and regulator of cell behavior, should be considered for efficient tissue regeneration. An ideal TE construct should synchronously simulate the biological activity and mechanical properties of the target tissue and properly integrate with the adjacent tissue to create a natural ECM‐like environment. Here, we provide an overview of various biochemical and biophysical triggers that affect cell adhesion, proliferation, differentiation, and cell–cell communication, as well as the associated cellular mechanisms and outcomes (Table 5).

**TABLE 5:** Different biochemical and biophysical cues in tissue engineering and their roles in various tissues

Factors			Roles in various tissues	References
Biochemical factors	Growth factors	EGF	Wound healing Proliferation and tensile strength of the dermis Epithelium differentiation	27, 28, 29, 30, 31, 32, 33, 34, 35, 36
		FGF	Morphogenesis Brain patterning Muscle regeneration RAS‐MAP and PI3K‐AKT activation Blood vessel formation Bone formation	37, 38, 39, 40, 41, 42, 43, 44, 45
		TGF‐β	Cell differentiation and proliferation Metabolism of ECM proteins Regulation of the inflammatory responses Stimulation of mesenchymal cells Inhibition of ectodermal cells Wound and tissue healing Autoimmune diseases suppression Matrix synthesis	10, 46, 47, 48, 49, 50, 51, 52, 53, 54, 55, 56
		BMP	Cell proliferation, migration, and differentiation Osteogenesis and cartilage formation Angiogenesis	52, 53, 54, 55, 56
		PDGF	Production of autocrine factors, fibronectin, and hyaluronic acid Osteogenesis and cartilage formation Vascularization	22, 57, 58, 59, 60, 61, 62, 63
		VEGF	Vascularization	64, 65, 66, 67, 68, 69, 70
		NTF	Cell growth, survival, migration and proliferation Re‐myelination Morphological plasticity Modulating intracellular signaling pathways Axonal regeneration and neural differentiation	71, 72, 73, 74, 75, 76, 77, 78, 79, 80, 81, 82
	Cytokines		Cell growth and differentiation Immunity regulation Regeneration of damaged tissue Bone regeneration Osteochondral regeneration	10, 14, 83, 84, 85
	ECM components		Cell survival, growth, adhesion, proliferation, and migration Wound repair Cartilage, bone, vascular, and skin regeneration	86, 87, 88, 89, 90, 91, 92, 93, 94, 95, 96, 97, 98, 99, 100, 101, 102, 103, 104, 105, 106, 107, 108, 109
	Polynucleotides	DNA	Stimulate transgene expression	120, 121, 122, 123, 124, 125
		RNA	Orchestrating tissue regeneration signaling pathways Gene transcription modification Posttranscriptional gene silencing Regulation of protein translation	126, 127, 128, 129, 130, 131, 132, 133, 134, 135, 136, 137, 138, 139, 140, 141, 142, 143
	Small molecules		Cell differentiation Endogenous stem cell lineage commitment Modulate specific intracellular processes Improve cell–cell and cell–scaffold interactions	144, 145, 146, 147, 148, 149, 150, 151, 152, 153, 154, 155, 156, 157, 158
	Herbal extracts		Cell differentiation Wound healing Bone regeneration Excellent antibacterial, antioxidant, and immunomodulatory effects Improve biocompatibility and biodegradability	156, 157, 158, 159, 160, 161, 162, 163, 164, 165, 166, 167, 168, 169, 170, 171, 172, 173, 174, 175, 176, 177
	Microbial derived compounds		Support proliferation and adhesion of cells Reduction of potential postoperative inflammation and infection Improve biocompatibility and biodegradability	178, 179, 180, 181, 182, 183, 184, 185, 186, 187
Biochemical factors	Surface topography		Cell proliferation and migration Rearrangement of cytoskeleton alignment and intracellular focal adhesion proteins Activation of intracellular signaling and mechano‐transduction pathways Chondrogenesis Osteogenesis Nutrient diffusion	203, 204, 205, 206, 207, 208, 209, 210, 211, 212, 213, 214, 215, 216, 217, 218
	Mechanical Cues		Cell quiescence, division, and differentiation Reorientation and reorganization of the cell cytoskeleton Tissue‐specific ECM components production Gene expression Cell protein profiling Lineage specification and commitment Osteogenesis Organogenesis	5, 87, 198, 219, 220, 221, 222, 223, 224, 225, 226, 227, 228, 229, 230, 231, 232, 233, 234, 235, 236
	Electrical cues		Regulating the function of receptors for different ions Neuron synapses Muscle contraction Osteogenesis Cardiogenesis Neurogenesis Wound healing	193, 202, 237, 238, 239, 240, 241, 242, 243, 244
	Low‐frequency magnetic fields		Improved cell alignment and proliferation Cytosolic Ca^2+^ and calmodulin activation Effective in musculoskeletal and neurological disorders Fracture healing Chondrogenesis Osteogenesis Neurogenesis	245, 246, 247, 248, 249
	Ultrasonic fields		Improved cell alignment and proliferation Cellular metabolism Applying energy to the cellular cytoskeleton Neuron conduction and differentiation Cardiomyocyte beating Regulate the cell cycle Activating ERKs/MAPKs pathways Fibroblast proliferation Angiogenesis Osteogenesis	250, 251, 252, 253, 254, 255
	Thermal energy		Killing osteosarcoma cells Myogenic and osteogenic differentiation	256, 257, 258, 259, 260, 261, 262
	Nonionizing, nonthermal light treatment		Cell survival Reducing inflammation Promoting tissue healing Osteogenesis Wound healing	263, 264, 265, 266, 267, 268, 269, 270, 271

Soluble biochemical inducers such as growth factors or cytokines are commonly used to regulate stem cell fate and tissue regeneration. Due to limitations in the use of biochemical factors, such as cost, off‐target activities, rapid degradation, and difficulty in determining optimal release kinetics, the focus has shifted to altering the biophysical properties of the cell microenvironment. Many studies have demonstrated the effects of biophysical factors such as electric and magnetic fields, mechanical forces, topographical features, and the use of electromagnetic, thermal, and ultrasonic energy on cell behavior. However, recent studies have shown that the simultaneous use of biochemical and biophysical signals can synergistically enhance the efficiency of TE approaches.

Remarkable progress has been made in the development of scaffold‐based platforms with well‐defined, spatially and temporally controlled physicomechanical signals. However, there are still some challenges and uncertainties that should be addressed for safer and more effective clinical implementation, such as controlling the dose of these factors to avoid adverse differentiation or cell transformation. Considering the dynamic extracellular environment that has viscoelastic properties, novel approaches focus on platforms with dynamic and tunable mechanical properties.

Many studies have shown that biochemical and biophysical cues can induce phenotypic changes in the cell. However, the subsequent changes in epigenetic state, including noncoding RNAs and DNA methylation, remain unclear. In this context, it is foreseeable that high‐throughput tools such as genomic, transcriptomic, epigenomic, and proteomic technologies could contribute significantly to determining the influence of the above factors on intracellular signaling pathways and gene expression at the single‐cell level.

With technological advances, the optimal conditions for the application of biophysical and biochemical factors could be simulated using advanced computational tools that simulate the relationship between cells and their extracellular matrices. Because any cellular change is likely to affect several intracellular pathways simultaneously, elucidation of the intracellular mechanisms underlying proliferation or differentiation is important not only to give us an overall picture of tissue biology but also to develop approaches to tissue regeneration and repair. In addition, understanding how the host immune system responds to the biochemical and biophysical cues is a new investigation in the field of TE to ensure the manipulation of cells in an immune‐modulated environment along with protection from inflammation.^236^

Despite the numerous studies and experiments published in this field and the large amount of knowledge and progress achieved in the last decades, the clinical implementation of TE strategies still faces many challenges, including regulatory and economic barriers.^324^ Indeed, there is ample room for further studies to address some critical questions about the wide range of inducing factors and their mutual effects. Clearly, cell behavior is very complex, so single factors have shown to be less efficient in cell stimulation than a combination of them.^325^ By and large, a thorough understanding of the role of the various biophysical and biochemical factors and their synergistic effects will increase the pace of research and experimentation to eventually achieve optimal scaffold design for TE.

## AUTHOR CONTRIBUTIONS

**Behnaz Bakhshandeh:** Conceptualization (lead); data curation (lead); project administration (lead); supervision (lead); writing – original draft (equal); writing – review and editing (lead). **Nika Ranjbar:** Conceptualization (equal); data curation (lead); writing – original draft (lead). **Ardeshir Abbasi:** Data curation (equal); writing – original draft (equal). **Elahe Amiri:** Data curation (equal); writing – original draft (equal). **Ali Abedi:** Data curation (equal); writing – original draft (equal). **Mohammad‐Reza Mehrabi:** Data curation (equal); writing – original draft (equal). **Zahra Dehghani:** Data curation (equal); writing – original draft (equal). **Cristian Pablo Pennisi:** Data curation, project administration, supervision, writing original draft, writing review editing.

## FUNDING INFORMATION

The authors received no specific funding for this work.

## CONFLICT OF INTEREST

The authors declare no conflicts of interest.

### PEER REVIEW

The peer review history for this article is available at https://publons.com/publon/10.1002/btm2.10383.

## Data Availability

Data sharing is not applicable to this article as no new data were created or analyzed in this study.
